# 18F-FDG Positron Emission Tomography/Computed Tomography in the Diagnosis and Post-Therapeutic Treatment in a Patient with an Early Stage of Retroperitoneal Fibrosis

**DOI:** 10.4274/Mirt.140

**Published:** 2013-08-01

**Authors:** Artor Niccoli Asabella, A Nicoletti, C Altini, A Notaristefano, G Lastilla, G Rubini

**Affiliations:** 1 University of Bari, Nuclear Medicine Unit – Di.M.I.M.P., Bari, Italy; 2 University of Bari, Anatomical Pathology Unit 2 – D.A.P.E.G., Bari, Italy

**Keywords:** 18F-FDG positron emission tomography/computed tomography, contrast enhancement computed tomography, idiopathic retroperitoneal fibrosis

## Abstract

Here, we report an experience about 18F-FDG-PET/CT in a patient with an early stage of Idiopathic Retroperitoneal Fibrosis (IRF). At the diagnosis Contrast Enhanced Computed Tomography (CE-CT) revealed periaortic solid tissue in the infrarenal section and locoregional lymph nodes; findings were interpreted as lymphomatous tissue. 18F-FDG-PET/CT showed elevated 18F-FDG uptake in the periaortic tissue but no uptake was detected in lymph nodes. The histologic examination showed recent-onset IRF. The patient began corticosteroid therapy. Nearly at the end of the therapy, CE-CT showed the enlargement of the fibrous tissue and 18F-FDG-PET/CT showed an increased 18F-FDG uptake in the aforesaid lesion and another area of uptake in the aortic wall. 18F-FDG-PET/CT can play an important role in the diagnosis of patients with an initial clinical suspicion of retroperitoneal fibrosis and in their management. Then the patient began a therapy with methotrexate and after six months we performed an 18F-FDG-PET/CT which didn’t show 18F-FDG uptake.

**Conflict of interest:**None declared.

## INTRODUCTION

Idiopathic Retroperitoneal Fibrosis (IRF) is a rare disease (incidence between 40 and 60 years; M:F=2:1), defined as a connective-tissue disorder, characterized by the development of fibro-inflammatory tissue in retroperitoneum (1,2).

It is an insidious disease, which could be initially silent, or characterized by non-specific and/or systemic symptoms, sometimes associated with local ones due to the entrapment of retroperitoneal structures. It mostly manifests itself locally through abdominal and back pain (1,2).

Here, we report an experience about ^18^F-FDG-Positron-Emission-Tomography/Computed-Tomography (^18^F-FDG-PET/CT) in the diagnosis and post-therapeutic evaluation in a patient with an early stage of IRF.

## CASE REPORT

**Figure 1:**
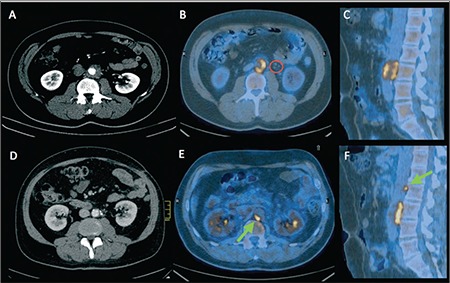
(A) Transaxial CE-CT image performed at diagnosis, showing periaortic solid tissue and locoregional lymph nodes. (B) Transaxial and (C) Sagittal 18F-FDG-PET/CT fused image performed at diagnosis, showing 18F-FDG uptake in the periaortic tissue; no 18F-FDG uptake in lymph nodes reported at CE-CT (red circle). (D) Transaxial CE-CT image performed during corticosteroid therapy, showing size reduction of the solid tissue. (E) Transaxial and (F) sagittal 18F-FDG-PET/CT fused image performed one month after the end of therapy, showing.

A 43-year-old man came at our attention for cramp-like abdominal pain not responding to anti-inflammatory or anti-spastic therapies. Blood and urine tests were normal. One month later a contrast-enhancement computed-tomography (CE-CT) revealed the presence of periaortic solid tissue in the infrarenal section (thickness: 14 mm; longitudinal extension: 46 mm; 48-73 HU) and locoregional lymph nodes (Figure 1A). 

These findings were interpreted as lymphomatous tissue. One week after CE-CT, blood test showed only increased erythrocyte sedimentation rate (ESR). One month after CE-CT, the patient underwent ^18^F-FDG-PET/CT in our Nuclear Medicine Unit, which showed elevated ^18^F-FDG uptake (Figure 1B-1C) in the periaortic tissue surrounding the descending aorta in front of the L3 vertebra (_SUV_max 4.6); no ^18^F-FDG uptake was detected in lymph nodes reported at the previous CE-CT (Figure 1B, red circle). After ^18^F-FDG-PET/CT the patient underwent laparoscopic biopsy; the histologic examination showed the presence of lobular lympho-plasmacellular aggregates and richly vascularized fibrous tissue, corresponding to recent-onset IRF. Then the patient began 9 months of corticosteroid therapy with Prednisone, with pain remission after the first month. After five months of therapy, the patient had a CE-CT (Figure 1D), which showed a size reduction of the solid tissue (thickness: 4 mm).

The inflammatory markers were normal. Nearly at the end of the therapy, corresponding with the lowering of prednisone dose, the patient reported the onset of abdominal pain. For this reason another CE-CT was performed which showed the enlargement of the fibrous tissue (thickness: 8 mm; longitudinal extension: 49 mm, 32-61 HU). A second ^18^F-FDG-PET/CT showed increased ^18^F-FDG uptake, corresponding to the aforesaid lesion (SUVmax 7.3). Another area of ^18^F-FDG uptake (Figure 1E-1F, green arrow) was observed on the right-posterior-lateral aortic wall, at L2 vertebra level (SUV max 5.1). Therefore, according to ^18^F-FDG-PET/CT findings, clinicians began a new regimen of prednisone and methotrexate (MTX). 

**Figure 2:**
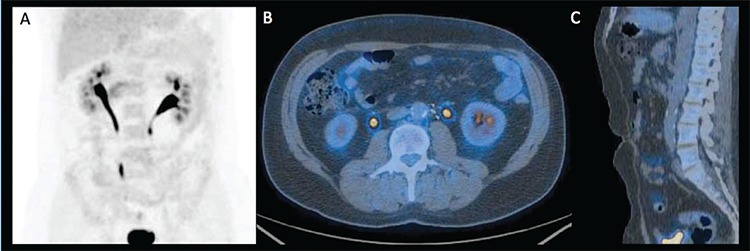
(A) MIP, (B) transaxial and (C) sagittal 18F-FDG-PET/CT fused image performed after 6 months from the beginning of therapy withprednisone and methotrexate which didn’t show 18F-FDG uptake.

After six months we performed an ^18^F-FDG-PET/CT which didn’t show any area of pathological ^18^F-FDG uptake, confirming the therapy’s outcome and then the end of the MTX use (Figure 2).Literature Review and DiscussionMacroscopically IRF appears as a fibrous mass, localized between the origin of the renal arteries and the bifurcation of the common iliac at the level of the L4-L5 vertebra (3). Histologically, in the early stages, the tissue is richly vascularized with chronic active inflammation (1,3); in advanced stages becomes less vascularized and richer in dense collagen fibers (1,4). CE-CT and magnetic resonance are the imaging techniques of choice for diagnosis and quantification of IRF. CE-CT shows a variable degree of enhancement: intense in the early and acute stages of the disease, minimal in advanced stages. ^18^F-FDG-PET/CT is used primarily to assess the metabolic activity of the mass. It is useful for identification of aortitis and periaortitis in the acute phase, evaluation of IRF after treatment and in cases of relapse (5,6). The first prospective study in 26 patients evaluated the potential role of ^18^F-FDG-PET/CT in assessing disease activity. It showed that positive ^18^F-FDG-PET/CT correlates with C-reactive protein (CRP) level and with lesion size. Visual PET score decreased after tamoxifen therapy and correlates with Erythrocyte Sedimentation Rate (ESR) reduction, but not with CT mass regression. The authors concluded that ^18^F-FDG-PET/CT is valuable in detecting disease activity (presumed IRF or suspected recurrence), particularly in the absence of symptoms and acute phase reactant increase (7). 

Definitive diagnosis requires biopsy (8) and histological examination. In medical therapy, steroids play a leading role (9). Methotrexate, azathioprine and mycophenolate are usually reserved for patients who do not benefit from corticosteroid treatments (10). Tamoxifen can also be used, due to its anti-inflammatory and anti-fibrosis action, but corticosteroid therapy is better than tamoxifen in maintenance of remission in patients with idiopathic retroperitoneal fibrosis, and induces a greater shrinkage of the retroperitoneal mass than tamoxifen does (11).

The peculiarity of this case is that our patient showed abdominal pain in an early phase because of the unusual higher location of the disease (at a L3 vertebra level, rich of nerve endings). 

In laboratory tests, the most important anomalies that occur are: increased ESR, CRP, the alpha-2-globulin (4). In our patient, CRP values were always within normal range, on the contrary, ESR values were variously amended in agreement with morphological data provided by CE-CT. 

^18^F-FDG-PET/CT led the initial differential diagnosis (lymphoma? Retroperitoneal fibrosis?) documenting the absence of glucose hyper-metabolism in the lymph nodes detected by CE-CT; it ruled out the presence of an extra-peritoneal localization of the disease and identified the most suitable site for biopsy. We did not perform an ^18^F-FDG-PET/CT during the therapy because the patient was asymptomatic, the inflammatory markers were normal and CE-CT showed lesion size reduction, a clear proof of a good response to therapy. ^18^F-FDG-PET/CT is able to identify with higher sensitivity compared to CT, the persistence of inflammation in the residual tissue after therapy (12,13).

In our patient, one month after the end of the therapy, ^18^F-FDG-PET/CT showed a higher ^18^F-FDG uptake than the previous ^18^F-FDG-PET/CT, despite ESR was altered still below the threshold of diagnosis; it also showed a new site of inflammation of the aortic vessel wall, not shown by the CE-CT. 

It was found interesting that CE-CT performed one month after the end of the corticosteroid therapy, compared to the CE-CT performed at the diagnosis, showed a size reduction by 43% and a CE reduction from 48-73 HU to 32-61 HU, while ^18^F-FDG-PET/CT, performed at the end of corticosteroid therapy, compared with that performed at the diagnosis, showed an increase of ^18^F-FDG uptake by 51%. We have hypothesized that metabolic changes that occurs in the recurrence precede the morpho-structural ones and justify the increment of ^18^F-FDG uptake and the absence of CE-CT increase at the same sites.

This metabolic evidence was important in the choice of the new therapeutic strategy. 

^18^F-FDG-PET/CT can play an important role in the diagnosis of patients with an initial clinical suspicion of retroperitoneal fibrosis, providing useful data for the differential diagnosis from other retroperitoneal diseases. It is also useful in cases of exacerbation and progression of the disease, highlighting new foci of inflammation, not documented at morphological imaging (CE-CT), helping the choice of a valid therapeutic strategy and monitoring the disease free survival.
